# Integrated time-serial transcriptome networks reveal common innate and tissue-specific adaptive immune responses to PRRSV infection

**DOI:** 10.1186/s13567-020-00850-5

**Published:** 2020-10-13

**Authors:** Byeonghwi Lim, Sangwook Kim, Kyu-Sang Lim, Chang-Gi Jeong, Seung-Chai Kim, Sang-Myeong Lee, Choi-Kyu Park, Marinus F. W. te Pas, Haesu Gho, Tae-Hun Kim, Kyung-Tai Lee, Won-Il Kim, Jun-Mo Kim

**Affiliations:** 1 Department of Animal Science and Technology, Chung-Ang University, Anseong, Gyeonggi-do 17546 Republic of Korea; 2 Department of Animal Science, Iowa State University, Ames, IA 50011 USA; 3 College of Veterinary Medicine, Jeonbuk National University, Iksan, Jeollabuk-do 54596 Republic of Korea; 4 College of Veterinary Medicine, Chungbuk National University, Cheongju, Chungcheongbuk-do 28644 Republic of Korea; 5 College of Veterinary Medicine & Animal Disease Intervention Center, Kyungpook National University, Daegu, 41566 Republic of Korea; 6 Wageningen UR Livestock Research, Wageningen, The Netherlands; 7 Animal Genomics and Bioinformatics Division, National Institute of Animal Science, RDA, Wanju, 55365 Republic of Korea

**Keywords:** adaptive immunity, gene co-expression network, innate immunity, integrated transcriptomes, porcine reproductive and respiratory syndrome virus

## Abstract

Porcine reproductive and respiratory syndrome virus (PRRSV) infection is the most important viral disease causing severe economic losses in the swine industry. However, mechanisms underlying gene expression control in immunity-responsible tissues at different time points during PRRSV infection are poorly understood. We constructed an integrated gene co-expression network and identified tissue- and time-dependent biological mechanisms of PRRSV infection through bioinformatics analysis using three tissues (lungs, bronchial lymph nodes [BLNs], and tonsils) via RNA-Seq. Three groups with specific expression patterns (i.e., the 3-dpi, lung, and BLN groups) were discovered. The 3 dpi-specific group showed antiviral and innate-immune signalling similar to the case for influenza A infection. Moreover, we observed adaptive immune responses in the lung-specific group based on various cytokines, while the BLN-specific group showed down-regulated AMPK signalling related to viral replication. Our study may provide comprehensive insights into PRRSV infection, as well as useful information for vaccine development.

## Introduction

Porcine reproductive and respiratory syndrome (PRRS) is one of the most important diseases affecting commercial pig productivity in the swine industry worldwide [1]. PRRS is caused by the PRRS virus (PRRSV), a single-stranded RNA virus [2, 3], resulting in severe reproductive losses for breeding pigs and respiratory problems for growing pigs [4]. Vaccination—a solution for this problem—is still limited because of the high mutation rate in the viral proteins and the intrinsic characteristics of PRRSV that impede innate immune responses [5–7]. Therefore, to date, several studies have been performed to identify host factors that confer resistance to PRRSV infection. Some mutations in the guanylate-binding protein 1 and cluster of differentiation 163 (*CD163*) genes were reported to be associated with PRRSV susceptibility [8, 9], and also CD163 knockout was proved to show full resistance for PRRSV infection [10].

Porcine alveolar macrophages (PAMs) represent the main cellular target for PRRSV infection [11]. PRRSV replication is initiated in the cytosol in infected cells following receptor-mediated endocytosis and disassembly, and replication primarily occurs in the lung and lymphoid organs, but not the spleen [12]. PRRSV can modulate host immune responses by down-regulating interferon-β production and suppressing the activity of antigen-presenting cells [13, 14]. In addition, serum viral loads have been found to increase for approximately 1 week after infection and then gradually decrease over the course of a month [15]. In contrast, viral loads in lymphoid organs are reported to reflect high viral replication for a few months [16].

Several recent studies have involved the use of RNA sequencing (RNA-Seq) to identify the functional basis of host responses to PRRSV infection. Serial blood transcriptomes observed following PRRSV infection in commercial pigs showed three main clusters related to immune signalling, DNA repair, and cell signalling [17]. Additionally, the blood transcriptomes in PRRSV-infected gilts indicated the development of innate immunity at 2 days post infection (dpi) and T cell signalling at 6 dpi [18]. Relatively few PRRSV studies in tissues based on RNA-Seq have been conducted thus far. The lung transcriptomes of PRRSV-infected pigs revealed genes that were potentially related to early innate immune responses [19]. Analysis of tracheobronchial-lymph node transcriptomic responses to highly pathogenic PRRSV infection revealed that they were mainly associated with cell death [20], and the tonsil transcriptomes of pigs revealed that high viral levels activated polarisation of blood cell functions [21]. However, in most studies, analysis was performed at one or two time points in a single tissue after PRRSV infection. Therefore, an examination of the dynamic regulatory changes in gene expression levels at serial time points in multiple tissues is needed to gain comprehensive insights into PRRSV infection.

In this study, we investigated the molecular mechanisms of PRRSV infection by integrating the differences in the expression of various genes in tissues responsible for respiration (lungs) and immunity (bronchial lymph nodes [BLNs], and tonsils) using RNA-Seq data at serial time points. Differentially expressed genes (DEGs) were identified at each time point and in each tissue after PRRSV infection. Then, dynamic molecular networks were constructed to identify tissue- and time-dependent gene expression levels and patterns.

## Materials and methods

### Cells and viruses

MARC-145 cells, which are highly permissive to PRRSV infection, were used for virus propagation and functional assays. MARC-145 cells were maintained in RPMI-1640 medium (Gibco® RPMI-1640, Life Technologies, Carlsbad, CA, USA) supplemented with heat-inactivated 10% foetal bovine serum (Life Technologies), 2 mM l-glutamine, and antibiotic–antimycotic (Anti-Anti, Life Technologies) containing 100 IU/mL penicillin, 100 µg/mL streptomycin, and 0.25 µg/mL amphotericin B in a humidified chamber at 37 °C under 5% CO_2_ conditions. The PRRSV-2 strain JA142 (GenBank: AY424271.1) was used in this study.

### Animals and sample preparation for RNA-Seq

**Figure 1:**
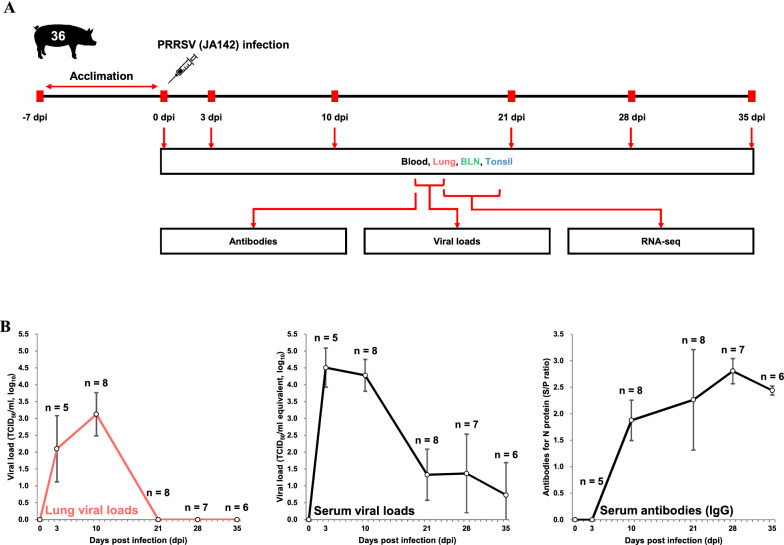
**Overview of the study design and the measured phenotypes.** **A** Schematic representation of the experimental design in terms of the sample types, target tissues (lungs, BLNs, and tonsils), and time points (0 dpi, 3 dpi, 10 dpi, 21 dpi, 28 dpi, and 35 dpi) after PRRSV infection. **B** Serum and lung viral loads and serum antibody levels in PRRSV-infected pigs.

4 weeks-old piglets (n = 40) were obtained from a PRRSV-negative farm and housed in animal rooms at our facility. After 7 days of acclimation, 34 pigs were intramuscularly challenged with 2 mL of PRRSV (JA142 strain; 1 × 10^3^ tissue culture infectious dose (TCID)_50_/mL), diluted in sterile PBS. All infected pigs were humanely euthanised at 3, 10, 21, 28, and 35 dpi, respectively. The remaining pigs were humanely euthanised without virus infection as an uninfected control (0 dpi) group. A schematic overview of the animal study is shown in Figure 1A. Blood was collected at 0, 3, 10, 21, 28, and 35 dpi from the euthanised pigs, and serum was separated for viral load detection and serological assays. The lungs, BLNs, and tonsils were aseptically extracted after euthanasia. These tissues were collected in tubes, snap-frozen using liquid nitrogen, and stored immediately at − 80 °C for RNA extraction. All animal experiments were approved by the Jeonbuk National University Institutional Animal Care and Use Committee, Republic of Korea (approval number 2016–43).

### Quantification of serum and lung viral loads

Viral RNA was extracted from 100 µL of each serum sample and 1 g of each tissue sample, using a MagMAX™ Viral RNA Isolation Kit (Ambion, Applied Biosystems, Life Technologies) and a total RNA Extraction Kit (Hybrid-RTM, GeneAll, Seoul, Republic of Korea), respectively, per the manufacturers’ instructions. Serum and lung viral loads were measured using a Prime-Q PCV2, PRRSV Detection Kit (GeNet Bio, Inc., Daejeon, Republic of Korea) with a 7500 Fast Real-Time PCR System (Applied Biosystems, Foster City, CA, USA). A standard curve was generated from known titres of PRRSV and used to calculate the quantity of PRRSV in each sample by converting each cycle threshold value to the TCID_50_/mL-equivalent values.

The PRRSV titres in lung tissues were measured with MARC-145 cells, using a microtitration-infectivity assay. Briefly, tissue homogenates [10% (weight/volume)] from the extracted lungs were prepared in Dulbecco’s modified Eagle’s medium with antibiotics, vortexed for 10–15 min, and centrifuged at ~ 4000 × *g* for 1 h at 4 ℃. After centrifugation, each collected supernatant was filtered through a sterile 0.20 μm syringe filter and incubated with MARC-145 cells to measure the viral titre. PRRSV titres were calculated at 5 to 6 dpi, based on the observed cytopathic effects, and were expressed as TCID_50_/mL.

### Detection of serum PRRSV antibodies

PRRSV-specific immunoglobulin G (IgG)-type antibodies were detected in the serum using a commercially available ELISA kit (Bionote PRRS Ab 4.0, Hwaseong, Republic of Korea) based on the detection of the nucleocapsid protein, according to the manufacturer’s instructions. The sample to positive (S/P) ratio of each serum sample was ≥ 0.4, which was considered to be indicative of the presence of PRRSV antibodies.

### RNA extraction, complementary DNA (cDNA) library construction, and RNA-Seq

Total RNA was extracted from the lung, BLN, and tonsil tissues using the TRIzol reagent (Invitrogen, Life Technologies) according to the manufacturer’s recommendations. Total RNA concentrations were calculated using Quant-IT RiboGreen (Invitrogen, Life Technologies, Carlsbad, CA, USA). To assess the RNA-integrity number, samples were run on the TapeStation RNA Screentape System (Agilent Technologies, Santa Clara, CA, USA) (Additional file 1). A cDNA library was independently prepared with 1 µg of total RNA for each sample using the Illumina TruSeq Stranded mRNA Sample Prep Kit (Illumina, Inc., San Diego, CA, USA). The first step in the workflow involved removing the rRNA from the total RNA, using the Ribo-Zero rRNA Removal Kit (Human/Mouse/Rat; Illumina, Inc.). Subsequently, the remaining mRNA was fragmented into small pieces using divalent cations under elevated temperature conditions. The cleaved RNA fragments were copied into first-strand cDNA using SuperScript II reverse transcriptase (Invitrogen, Life Technologies) and random primers. This step was followed by second-strand cDNA synthesis using DNA Polymerase I, RNase H, and dUTPs. The cDNA fragments were subjected to an end-repair process, involving the addition of a single ‘A’ base, after which adapters were ligated. The products were then purified and enriched by PCR to create the final cDNA library. The libraries were quantified using KAPA Library Quantification kits for Illumina Sequencing platforms, according to the qPCR Quantification Protocol Guide (Roche, Basel, Switzerland), and the libraries were validated using the TapeStation D1000 ScreenTape System (Agilent Technologies, Santa Clara, CA, USA). The indexed libraries were then analysed on an Illumina HiSeq4000 instrument (Illumina, Inc.), and paired-end (2 × 100 base pair) sequencing was performed. All raw RNA-Seq data generated in this study were deposited in the NCBI Sequence Read Archive database under the accession number PRJNA640269.

### Data processing and DEG analyses

To select the quality-filtering strategy, a quality check of raw read data was performed for each sample using the FastQC software v0.11.7, and the reads were trimmed with adaptors using the Trimmomatic software v0.38 based on the quality results. Then, the trimmed reads were re-checked with FastQC and mapped to the reference genome (*Sus scrofa* 11.1, GCA_000003025.6) of the Ensembl genome browser (https://www.ensembl.org/Sus_scrofa/) as the default option of the HISAT2 v2.1.0 programme. Raw counts corresponding to the genes in each library were calculated based on the exons in *Sus scrofa* GTF v95 (Ensembl) as the genomic-annotation reference file, using the featureCounts of Subread package, v1.6.3. All DEG analyses for the obtained raw counts were performed using the edgeR software package v3.26.5 of Bioconductor. To reduce statistical bias in the DEG analyses, genes were excluded when all samples had raw counts of ≤ 10. Normalisation of the raw counts was performed using the trimmed mean of M-value (TMM) method, and dispersion parameters were estimated and applied using the Cox–Reid profile-adjusted likelihood method in edgeR. DEGs were identified for each time point (3, 10, 21, 28, and 35 dpi; versus gene expression at 0 dpi) for each tissue (lungs, BLNs, and tonsils) using a negative binomial-generalised linear model, and *P*-values were corrected for multiple comparisons based on the false discovery rate (FDR). DEGs were determined based on an FDR of < 0.05 and an absolute log_2_ fold-change (FC) of ≥ 1. Multidimensional scaling (MDS) was performed using the limma function of the R package to identify the similarities among samples.

### Gene co-expression network (GCN) and clustering analyses

GCN analysis was conducted by filtering out: (i) DEGs with no significant FDR (FDR < 0.05) observed at any of the 5 time points in 3 tissues and (ii) DEGs with a not stringent significant value (absolute log_2_ FC ≥ 2.0), in order to increase the efficiency of network construction. Before GCN analysis, significant associations between the filtered genes were calculated using the partial correlation coefficient with information theory (PCIT) algorithm [22]. Correlations were estimated to assess co-expression, and the network was constructed using genes with absolute co-expression correlations of ≥ 0.90. GCN visualisation was performed using the Cytoscape v3.7.1 software, and the resulting network consisted of genes (nodes) and connections (edges).

Clustering analysis was performed using the log_2_ FC values of genes in the constructed network. After determining the optimal number of clusters, the genes were analysed using the k-means clustering algorithm with 1000 iterations, using the Multi Experiment Viewer (MeV) software.

### Functional analyses

The genes in the constructed GCN were classified as up- and down-regulated genes based on the time point for tissue that showed the maximum FC, and were annotated to the Kyoto Encyclopaedia of Genes and Genomes (KEGG) using Database for Annotation, Visualization and Integrated Discovery (DAVID) v6.8. In addition, enrichment analyses were performed with BPs, using Gene ontology (GO) terms and KEGG pathways for the genes in each GCN. GO annotations were filtered with the DIRECT option and applied to enrichment analyses with the following cut-offs: *P* value < 0.1 and counts ≥ 2. Next, treemaps for the enriched GO terms were visualised using the REVIGO tool. KEGG annotations were also enriched using the same cut-off criteria and are represented by the –log_10_ *P* value and fold enrichment. All data used in the enrichment analyses were annotated in *Sus scrofa*.

### Gene set-enrichment analyses (GSEA) and protein–protein interaction (PPI) network analysis

GSEA for GCN group-specific genes were conducted using the gene-ranking method based on gene sets in the KEGG database to determine the enrichment scores and statistically significant differences, using the GSEA v4.0.2 software. All analyses were performed using the log_2_-normalised TMM counts of the selected tissues and time points that showed the largest expression changes in each group. Counts corresponding to: (i) 3 dpi in all tissues were used for the 3 dpi-specific group, (ii) 10 dpi in lung tissues were used for the lung-specific group, and (iii) 10 and 35 dpi in BLN tissues were used for the BLN-specific group. GSEA results were visualised as enrichment maps with significant pathways (FDR < 0.05) following the Benjamini–Hochberg correction using Cytoscape, and their connections indicated the similarities between gene sets. Additionally, the core enriched genes of pathways showing the highest normalised enrichment score (NES) were expressed as heatmaps. Then, the modulations of responsible gene products (proteins) in the selected representative KEGG pathways (determined through GCN and GSEA) were confirmed using the clusterProfiler package in the R software. Among the genes corresponding to each protein, genes showing the maximum changes were used as the representative values. The PPI network was investigated for the top 25 interactions (identified in this study) with extremely high gene expression levels using the *Homo sapiens* database of STRING v11.0.

## Results

### PRRSV loads and antibodies

Serum and lung viral loads and serum antibody levels at each time point after PRRSV infection are shown in Figure 1A–B. The lung viral loads were comparatively lower than the serum viral loads at all time points (*p* = 0.0015). The viral loads in the serum and lung samples were highly correlated (*r* = 0.83), showing the highest values during the early stages of infection (3 and 10 dpi). The viral loads decreased markedly beyond 21 dpi, and lung samples without detectable viral loads were observed during these time points. Serum antibodies (IgG) were first detected at 10 dpi and their levels increased up to 28 dpi, but slightly decreased at 35 dpi.

### Overview of data processing and integration of immunity-responsible tissue transcriptomes

A total of 4.3 billion paired-end sequence reads were produced from 120 tissue samples (from 3 tissues of 40 individuals), and the average of the number of reads produced per sample was 35.6 million (Additional file 1). The reads that passed the trimming process were mapped to pig reference genome 11.1 (~ 97.82% identity), and the average unique mapping rate was 87.47%, ranging from 81.10 to 91.62%.

**Figure 2:**
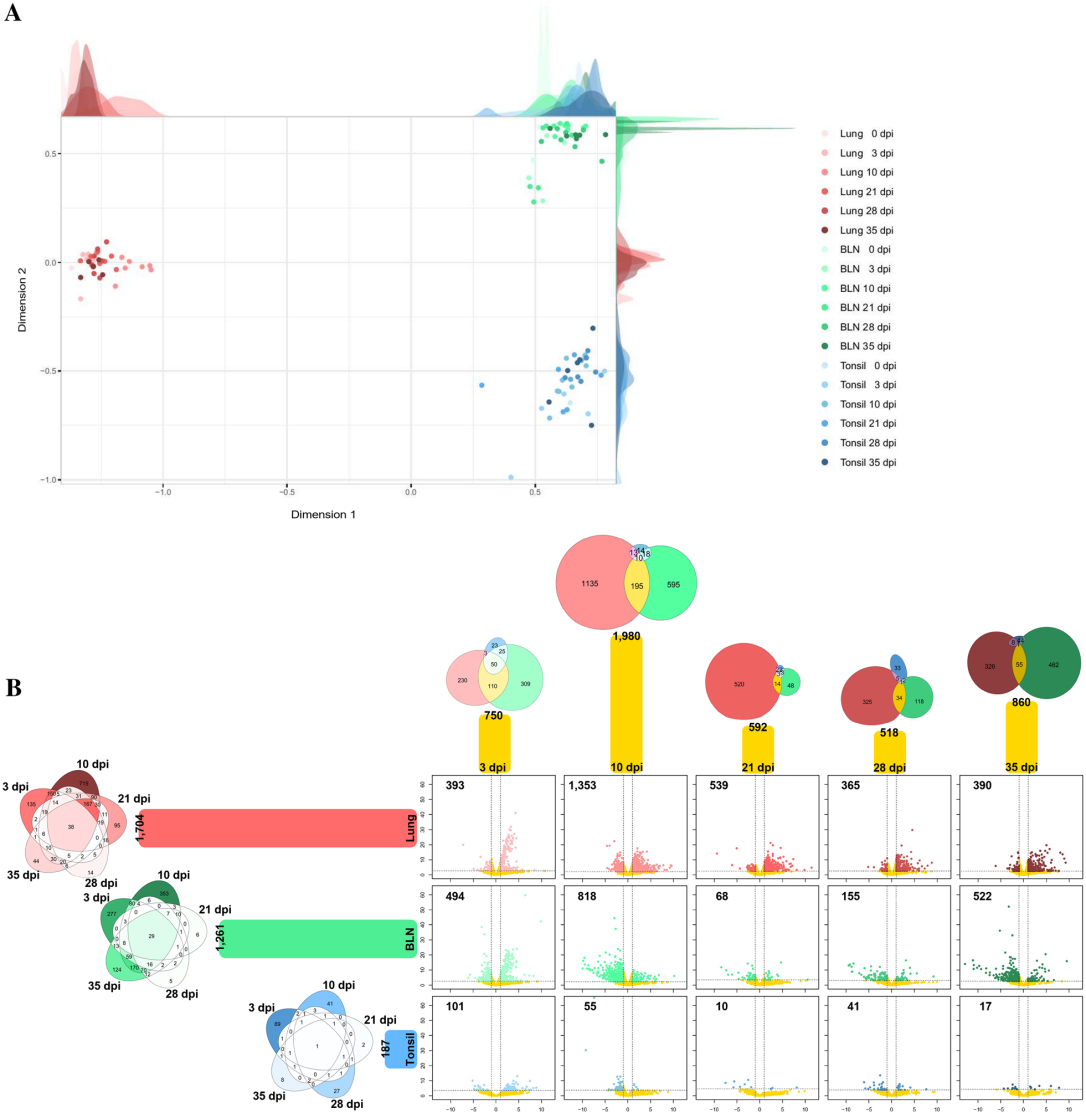
**Transcriptomes in respiratory and immune tissues after PRRSV infection.** **A** MDS revealed separate clusters among the three tissue groups, based on the transcriptomes at six time points after PRRSV infection. **B** Dynamic view of DEGs based on the starting time point (0 dpi) of PRRSV infection. The x and y axes of the volcano plots show the log_2_ FCs and − log_10_ *P* values, respectively. Data information: Significant DEGs (FDR < 0.05 and absolute log_2_ FC ≥ 1) are represented in the volcano plots, with the three different colours (red, green, and blue) corresponding to each tissue type, and the numbers of DEGs are written in the top left corner of each plot. Volcano plots and Venn diagrams for different time points are indicated with colour gradients. Tissue-specific Venn diagrams are illustrated with a colour gradient, based on overlapping numbers of DEGs at serial time points. Venn diagrams with scale bars show the numbers of integrated DEGs observed at each time point for each tissue.

Transcriptome read data were produced using 3 tissues (lungs, BLNs, and tonsils) at 6 time points (0, 3, 10, 21, 28, and 35 dpi), as shown in Figure 1A. The transcriptomes produced under PRRSV infection showed clear clustering for each tissue type, as determined by MDS analysis (Figure 2A). DEGs were confirmed by comparing the gene expression levels at each time point (3, 10, 21, 28, and 35 dpi), relative to those at 0 dpi, and overlapping DEGs among tissues and different time points are shown in a Venn diagram (Figure 2B). We observed dynamic changes in the gene expression levels in lung and BLN tissues at each time point. Lung tissues demonstrated a large proportion of up-regulated genes at all time points (3 dpi: 83%, 10 dpi: 62%, 21 dpi: 81%, 28 dpi: 86%, and 35 dpi: 83%) against 0 dpi, whereas BLN tissues mostly displayed a large proportion of down-regulated genes (3 dpi: 41%, 10 dpi: 89%, 21 dpi: 76%, 28 dpi: 72%, and 35 dpi: 86%). Interestingly, we observed a tendency towards gene up-regulation at 3 dpi in all tissues. Moreover, the numbers of DEGs in lung and BLN tissues increased markedly at 10 dpi, then sharply decreased, and slightly increased at 35 dpi. Tonsil tissues showed relatively subtle changes; therefore, we had difficulties in analysing them at all time points, except at 3 dpi (101 genes).

### GCN analysis following PRRSV infection

**Figure 3:**
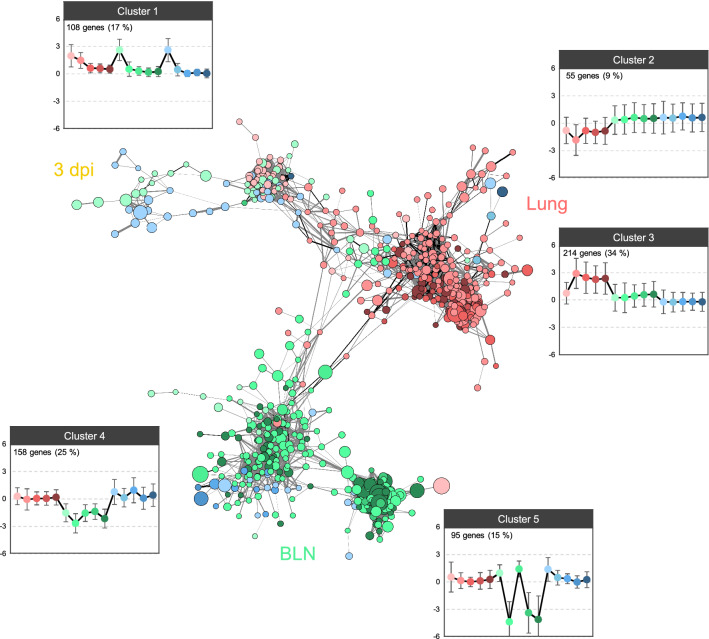
**A GCN was constructed following PRRSV infection, indicating three substantial core groups (3 dpi, lungs, and BLNs).** Each node (gene) colour is separated according to the associated immunity-responsible tissues: lungs (red), BLNs (green), and tonsils (blue). Nodes corresponding to each tissue were colour-coded using a gradient, based on each time point. The PCIT algorithm produced significant connections, and a titular threshold (${r}^{2}$>0.90) was used for the network analysis. The entire GCN contained 630 nodes connected by 7565 edges. The node size was mapped to the highest log_2_ FC value among all time points and all tissues. The edge widths represent the correlation coefficient between nodes, and the edge colours indicate whether the correlation coefficient had a positive (grey) or negative (black) value. Gene-clustering analysis using the k-means clustering algorithm in the MultiExperiment Viewer software revealed five clear expression patterns at different time points (left to right) and in different tissues (same colour as the node). The y axes in the graphs showing the clustering analysis represent the log_2_ FC values. The number of genes and the proportions of each cluster are shown in the top left corner.

GCN construction using 630 genes and 7565 significant connections (selected using the PCIT algorithm) was performed to integrate the transcriptomes for one respiratory and two immunity-related tissues at all time points (Figure 3). The locations of genes were marked close to one another when many common neighbours were found. The resulting network was precisely concentrated for three groups (3 dpi, lung tissue, and BLN tissue), and the groups were linked by some genes. Five clusters were identified via clustering analysis using the log_2_ FC values of genes in the GCN and were specifically matched to each group. Cluster 1, representing the 3 dpi-specific group, was composed of 108 genes that were up-regulated in all tissues with PRRSV at 3 dpi. Clusters 2 and 3, which represented the lung-specific group, contained 55 down-regulated genes at 10 dpi and 214 up-regulated genes at 10–35 dpi in the lungs, respectively. The BLN-specific group comprised two clusters of strongly down-regulated genes (158 genes in cluster 4 and 95 genes in cluster 5) at 10 and 35 dpi in BLN tissues. Analysis of cluster 5 showed that the genes were slightly up-regulated at 3 and 21 dpi in BLNs. Additionally, the BLN-specific group contained some genes that showed maximum changes in the tonsils.

**Figure 4:**
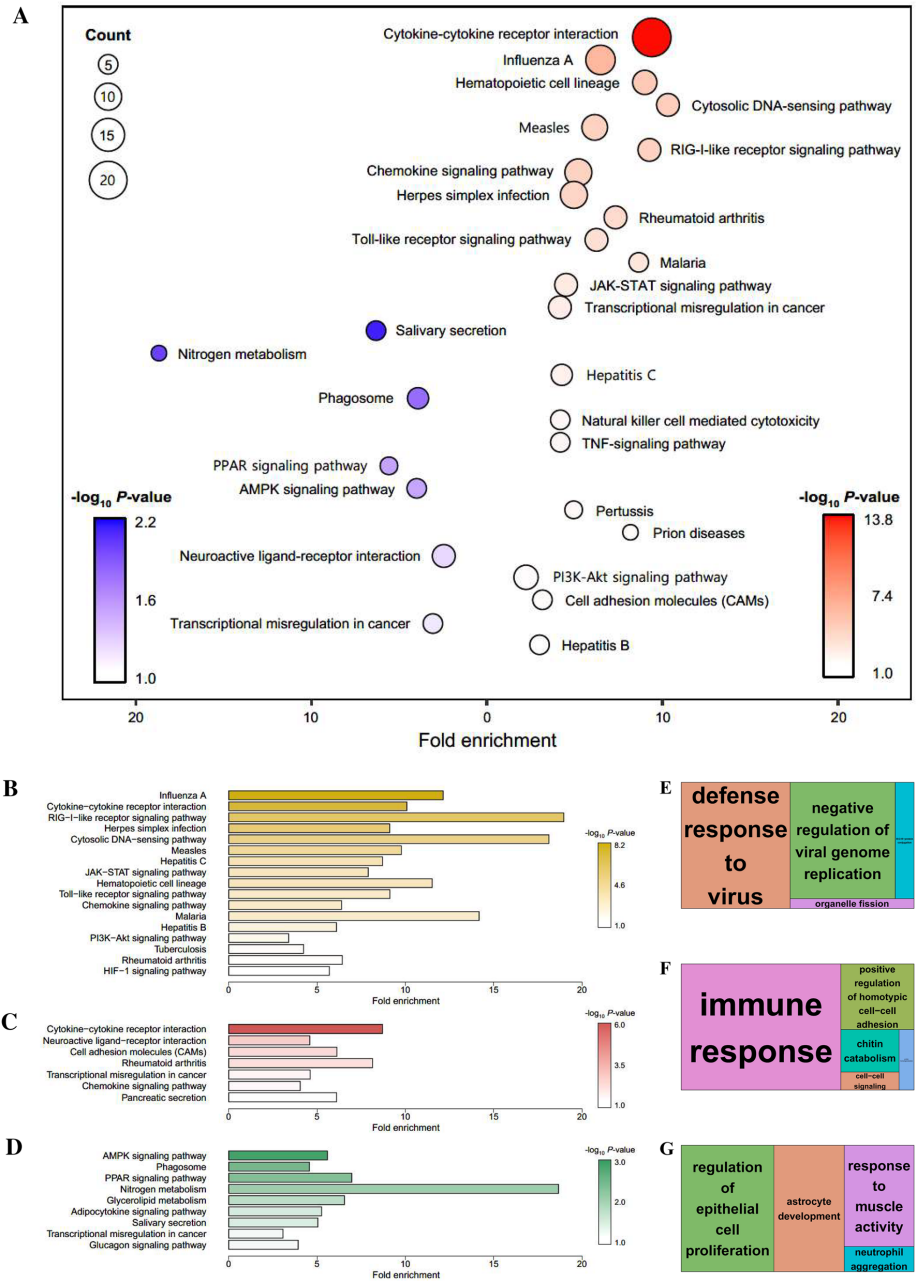
**Enrichment analyses based on the DAVID database, aimed at discovering the biological meaning of representative up- and down-regulated signalling pathways following PRRSV infection.** **A–G** Whole GCN network (**A**), 3 dpi-specific network (cluster 1; **B** and **E**), lung-specific network (clusters 2 and 3; **C** and **F**), and BLN-specific network (clusters 4 and 5; **D** and **G**) were used for the analyses. KEGG-enriched pathways for the whole GCN network (**A**) and each specific network (**B**–**D**) were visualised by generating bubble and bar plots. A bubble plot corresponding to the whole network was generated for each analysis for the up-regulated (red) and down-regulated genes (blue) (**A**). Data information: Significantly enriched pathways represented in the plots met the following cut-off criterion: − log_10_ *P* value > 1.0. GO treemaps were created based on the *P* values associated with the BP terms for each specific network (**E**–**G**).

We selected representative up- and down-regulated genes at different time points in different tissues with relatively high absolute FC values in the constructed GCN and categorised them as follows: 343 up-regulated genes and 287 down-regulated genes; significant pathways were identified by performing KEGG enrichment analyses using DAVID (Figure 4A). The bubble plot of the enriched biological pathways revealed up-regulated genes in KEGG pathways that are mainly related to immune responses (i.e., cytokine–cytokine receptor interaction, influenza A, cytosolic DNA-sensing pathway, and RIG-I like receptor signalling pathways) and down-regulated genes in pathways mainly related to energy metabolism (i.e., nitrogen metabolism, PPAR signalling pathway, and AMPK signalling pathway). These pathways provided important insights into the specific immune responses of host cells to PRRSV infection.

### Functional annotations for specific groups

Functional-enrichment analyses based on the KEGG (Figure 4B–D) and GO (Figure 4E–G) databases were performed to investigate the biological processes (BPs) associated with each group following PRRSV infection. The KEGG pathways of the 3 dpi-specific group were enriched for terms related to viral infection, including influenza A, the RIG-I-like receptor signalling pathway, herpes simplex infection, and the cytosolic DNA-sensing pathway (Figure 4B). In the lung-specific group, KEGG terms associated with immune responses, such as cytokine–cytokine receptor interaction, rheumatoid arthritis, and the chemokine signalling pathway, were identified (Figure 4C). Interestingly, the BLN-specific group showed KEGG terms related to lipid metabolism such as the AMPK signalling pathway, the PPAR signalling pathway, glycerolipid metabolism, and the adipocytokine signalling pathway (Figure 4D). The illustrated treemaps revealed BPs for significant GO terms such as defence response to virus and negative regulation of viral genome replication in the 3 dpi-specific group (Figure 4E); immune response in the lung-specific group (Figure 4F); and regulation of epithelial cell proliferation in the BLN-specific group (Figure 4G). The enrichment results were consistent between the KEGG and GO terms.

### GSEA and gene modulations associated with the mechanisms of host responses to PRRSV infection

**Figure 5:**
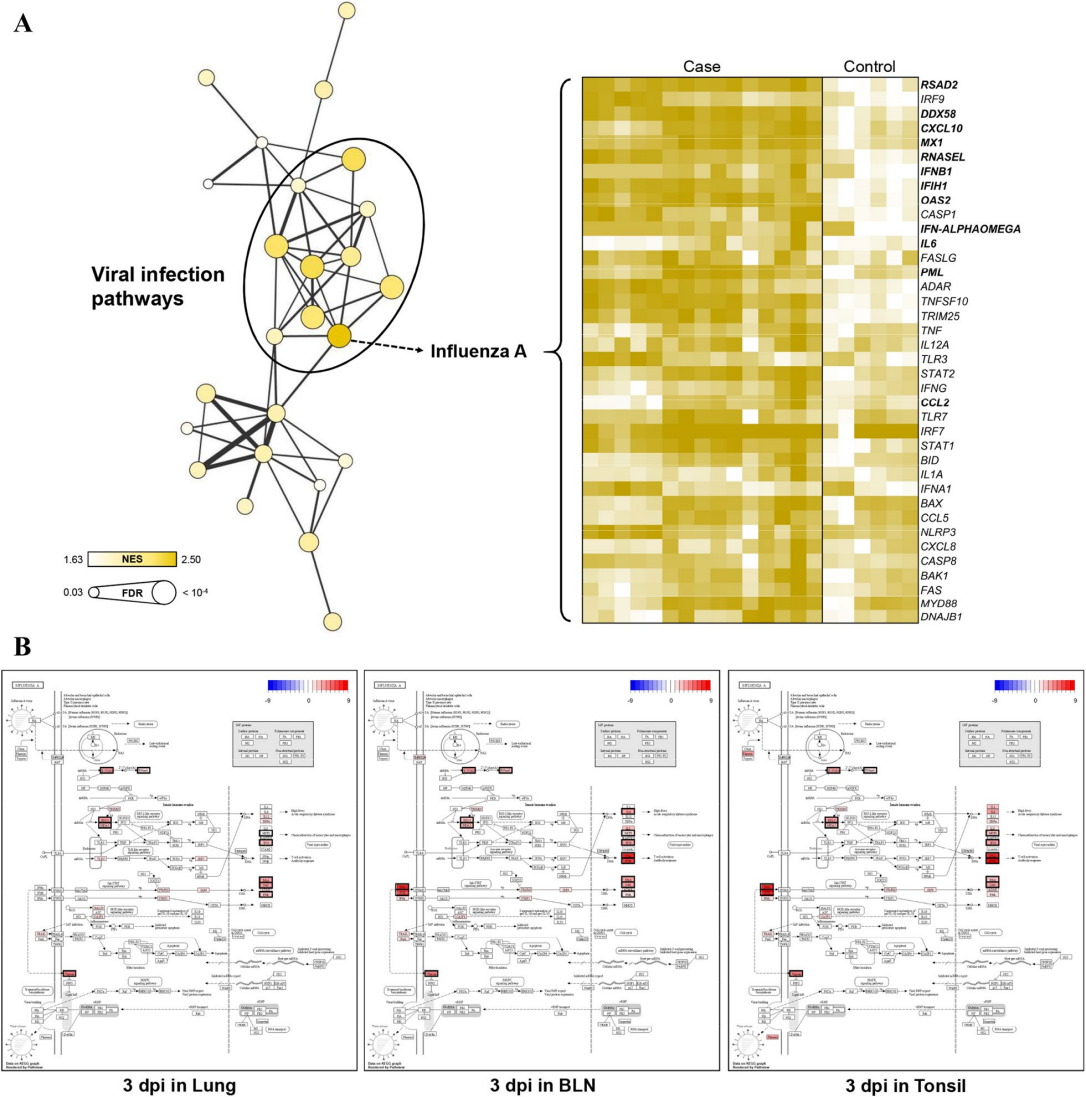
**GSEA results and gene modulations related to KEGG pathways at 3 dpi.** **A** An enrichment map (node: pathway, edge: similarity coefficient between gene sets) was built based on significant pathways (FDR < 0.05), and a heatmap was generated using the core enriched genes in the pathway with the highest NES (influenza A). **B** Genes overlapping with the GCN are shown in bold. Expression changes at 3 dpi in the influenza A pathway are presented as log_2_ FC values for each tissue. Data information: Proteins corresponding to the DEGs involved in the GCN are indicated with a black border.

**Figure 6:**
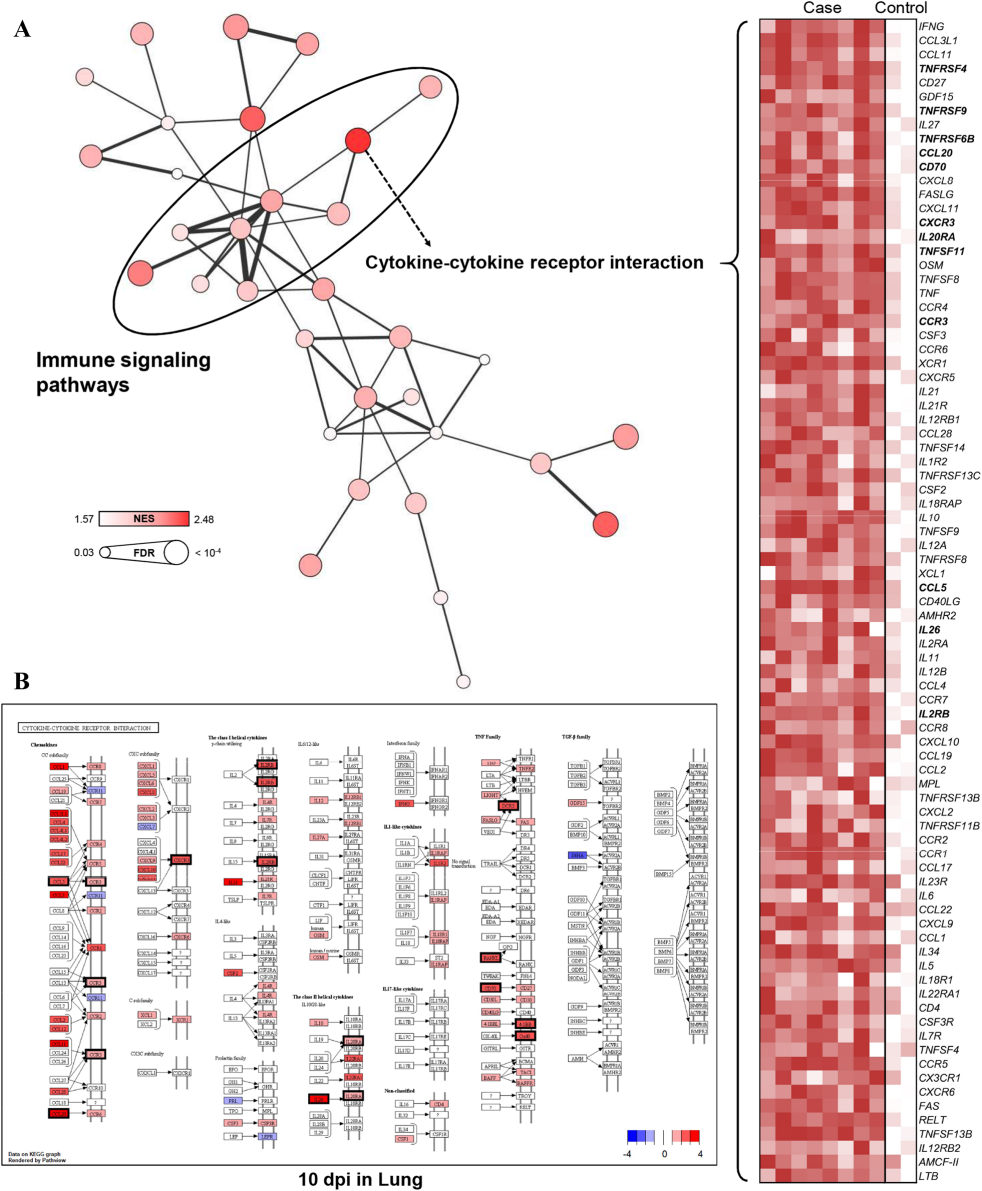
**GSEA results and gene modulations related to KEGG pathways at 10 dpi.** **A** An enrichment map (node: pathway, edge: similarity coefficient between gene sets) was built based on the significant pathways (FDR < 0.05), and a heatmap was generated using the core enriched genes in the pathway with the highest NES (cytokine–cytokine receptor interaction). **B** Genes overlapping with the GCN are shown in bold. Expression changes at 10 dpi in the cytokine–cytokine receptor interaction pathway are presented as log_2_ FC values in lung tissues. Data information: Proteins corresponding to DEGs involved in the GCN are indicated with a black border.

To validate the results of specific groups, GSEA based on the KEGG database were performed using log_2_ -normalised TMM counts at each time point for each tissue to identify the maximum gene expression changes for each group, and the GSEA results were consistent with the KEGG enrichment analyses of DEGs. GSEA using the 3-dpi data for all tissues revealed many significant pathways related to viral infection, among which influenza A showed the highest NES (Figure 5A). The expression levels of 38 core enriched genes in influenza A were visualised by generating a heatmap, and 12 (*RSAD2*, *DDX58*, *CXCL10*, *MX1*, *RNASEL*, *IFNB1*, *IFIH1*, *OAS2*, *IFN-ALPHAOMEGA*, *IL6*, *PML*, and *CCL2*) out of the 38 genes were included in the GCN. Studying the modulation of host genes related to influenza A infection at the 3-dpi time point for all tissues identified several linked genes (proteins) including *RSAD2* (Viperin), *DDX58* (RIG-I), *CXCL10* (IP10), *MX1* (MxA), *RNASEL* (RNaseL), *IFIH1* (MDA5), *OAS2* (2′-5′OAS and OAS), and *PML* (PML), and lymphoid organs (BLNs and tonsils) showed specific gene modulations for *IFNB1* (IFNβ), *IFN-ALPHAOMEGA* (IFNα), *IL6* (IL6), and *CCL2* (MCP1) (Figure 5B). GSEA using the 10-dpi data for lung tissues revealed many significant pathways related to immune responses, among which the cytokine–cytokine receptor interaction pathway showed the highest NESs (Figure 6A). The expression levels of 83 core enriched genes involved in cytokine–cytokine receptor interactions were visualised by generating a heatmap, and 12 (*TNFRSF4*, *TNFRSF9*, *TNFRSF6B*, *CCL20*, *CD70*, *CXCR3*, *IL20RA*, *TNFSF11*, *CCR3*, *CCL5*, *IL26*, and *IL2RB*) out of 83 genes were included in the GCN. Studying the modulation of genes related to cytokine–cytokine receptor interactions at the 10-dpi time point for lung tissues identified several genes (proteins) including *TNFRSF4* (Ox40), *TNFRSF9* (4-1BB), *TNFRSF6B* (DCR3), *CCL20* (CCL20), *CD70* (CD70), *CXCR3* (CXCR3), *IL20RA* (IL20RA), *TNFSF11* (RANKL), *CCR3* (CCR3), *CCL5* (CCL5), *IL26* (IL26), and *IL2RB* (IL2RB) (Figure 6B). The GSEA results obtained with the 10- and 35-dpi data for the BLN tissues could not be used to construct an enrichment map because only a few statistically significant pathways and low similarity were observed among the gene sets.

**Figure 7:**
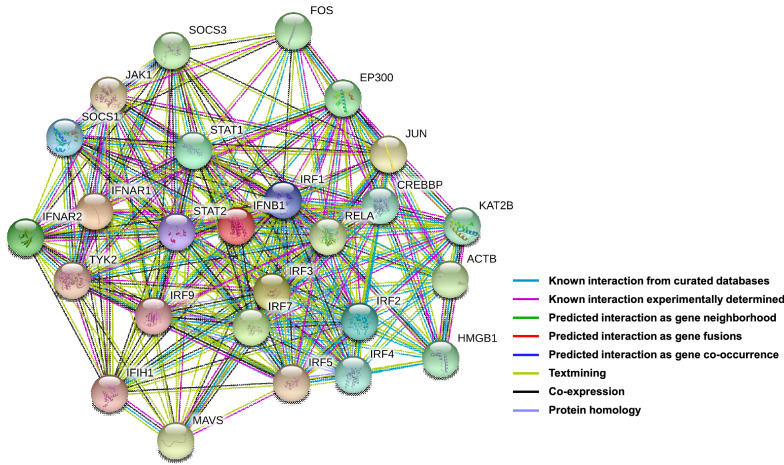
**PPIs depicted based on IFNB1 using the STRING database.** The top 25 proteins are shown.

In the PPI network, the top 25 proteins (i.e., those with the strongest interactions with *IFNB1* (IFNB1), and the largest differences in expression levels) were all immune-related proteins (IFNAR1, IRF3, IRF7, IFNAR2, STAT1, IRF2, SOCS1, IRF1, STAT2, IRF9, IFIH1, TYK2, IRF5, JAK1, JUN, MAVS, RELA, ACTB, SOCS3, FOS, EP300, HMGB1, KAT2B, CREBBP, and IRF4) (Figure 7). The interaction values are shown in detail in Additional file 1.

## Discussion

### Serial DEGs observed among tissues after PRRSV infection

PRRSV infection in pigs can cause a complicated disease when functioning as a primary respiratory infectious agent or as a cofactor in porcine respiratory disease complex (PRDC), and PRRSV was reported to be the most common virus associated with PRDC [23–26]. In addition, PRRSV was reported to inhibit the host immune defence system, which can lead to further infections (secondary/opportunistic pathogens), resulting in more serious and chronic diseases [12, 27]. Therefore, understanding the functional and regulatory mechanisms of respiratory and immunity-responsible tissues in the host during PRRSV infection is essential for preventing and controlling diseases directly linked to animal productivity.

In this study, we compared and integrated serial whole transcriptomes for three tissues (lungs, BLNs, and tonsils) during PRRSV infection. Interestingly, the lungs had lower viral loads than the serum (Figure 1B), even though the lungs showed major symptoms during PRRSV infection [28]. Based on MDS analysis, a well-defined trajectory of transcriptomes related to PRRSV infection was identified in each tissue (Figure 2A). The number of DEGs increased until 10 dpi, then decreased until 28 dpi, and finally increased slightly at 35 dpi (Figure 2B). The drift curves for the DEGs were very similar to serum and lung viral load curves, confirming that the overall host responses according to PRRSV infection may be most active at approximately 10 dpi, although they were not comparable to the serum antibody levels (Figure 1B). Previous reports showed that viral loads were detectable in lymphoid organs (excluding the spleen) until immediately before viral extinction [12, 16], although no similarity was reported in terms of the number of identified DEGs in this study.

### Dynamic changes in gene expression levels after PRRSV infection

PRRSV can infect cells of the macrophage and monocyte lineages in pigs, and thus, it may affect many aspects of tissue remodelling, development, immunity, and pathology [29]. PRRSV can delay innate and adaptive immune responses by inhibiting the production of type-I interferons (IFNs), especially IFN-α, which is important for intracellular signal transduction [27, 30]. Because the lungs are known as the main target of PRRSV infection and lymphoid organs can serve as viral reservoirs [31], the observation of common DEGs probably indicates identical host responses among these tissues, whereas unique DEGs for each tissue may serve specific functions in the respective tissues during PRRSV infection.

Weighted GCNs, which indicate specific gene expression changes, have been used as a powerful approach for identifying specific molecular mechanisms at the system level [32]. We integrated the transcriptomes of multiple tissues and time points by constructing a GCN with stringent DEGs and a PCIT algorithm (Figure 3). The constructed network revealed a clear separation of one time point group (3 dpi), one respiratory responsible tissue group (lungs), and one immunity-responsible tissue group (BLNs), and each group showed dynamic changes in the host-response system after PRRSV infection. In addition, the genes included in the lung and BLN groups showed specific differential expression in each tissue, whereas the genes included in the 3-dpi group were commonly expressed in all tissues. These features suggest that host responses to PRRSV infection are mainly regulated through the expression of different genes in the lungs and lymphoid organs (BLNs and tonsils). Furthermore, gene expression levels may be similarly regulated at 3 dpi in three tissues. Dynamic changes in these significant gene subsets at 3 dpi may represent biological signals associated with general and early immunological mechanisms in response to virus infection.

### Common initial innate immune responses to PRRSV infection

Significant KEGG terms related to viral infections and immune signalling were identified through KEGG enrichment analyses performed for each up- and down-regulated genes (343 and 287 genes, respectively), based on the time point for each tissue with a maximum FC value in the GCN, which was constructed using serial DEGs (Figure 4A). In particular, the enriched terms related to RNA viral infection (influenza A, the RIG-I-like receptor signalling pathway, measles, hepatitis C virus (HCV), and the Toll-like receptor signalling pathway) and innate immune signalling (cytokine–cytokine receptor interaction, the JAK-STAT signalling pathway, haematopoietic cell lineage, and the chemokine signalling pathway) were found among the highly significant pathways in the 3 dpi-specific group (Figure 4B). In addition, GSEA results based on the expression levels at 3 dpi in all tissues revealed clustering with KEGG terms related to viral infection, and influenza A showed the highest NES (Figure 5A). Previous reports showed that RIG-I-like receptors (RLRs), consisting of three proteins (RIG-I, MDA5, and LGP2), are one type of pattern-recognition receptors (PRRs) that activate innate immune-signalling pathways by detecting viral RNA in the cytosol [33, 34]. Influenza A viruses are RNA viruses that express non-structural protein 1 (NS1), which is important for evading toxic innate immune responses. NS1 can suppress mRNA processing and transport, which inhibits the binding of the viruses to double-stranded RNA molecules and restrains RLR activation [35–37]. These functions are known to interfere with, and delay, both the expression of the cytokine IFN-α/β and the initiation of an IFN-induced antiviral state [38]. It has been reported that both the structural and non-structural proteins of PRRSV have polygenic toxicity in hosts [39], particularly, nsp2TF and nsp2N generated by a ribosomal frameshift mechanism affect the suppression of cellular innate immune responses [40]. These features of PRRSV can be very susceptible to IFN-α/β, although they do not exhibit typical innate immune-signalling activation, including type-I IFN responses [41]. In addition, signalling associated with the RIG-I-like receptor and JAK-STAT pathways, which play important roles in IFN production, can become disrupted by PRRSV during acute infection [30]. Additionally, analysing the lung transcriptomes of PRRSV (North American strain CH 1a)-infected pigs (Landrace $\times$ Yorkshire) showed DEGs associated with inflammatory signalling at 4 dpi [19], which is similar to our results. Thus, the enriched biological terms for the 3 dpi-specific group found in this study suggest that both PRRSV and influenza A virus, both of which cause respiratory illnesses, exhibit similar infection mechanisms, immune evasion, and innate immune signalling. Additionally, the RIG-I-like receptor is thought to function as the main PRR for PRRSV during early immune responses.

### Adaptive immune responses to PRRSV infection in the lungs

KEGG enrichment analysis of the lung-specific group (containing clusters 2 and 3 of the GCN) implicated cytokine–cytokine receptor interactions, cell-adhesion molecules, rheumatoid arthritis, and chemokine signalling pathways in PRRSV infection, all of which are related to immune signalling (Figure 4C). Cluster 3 included several genes related to immune responses, which were especially up-regulated at 10–35 dpi in the lungs (Figure 3). GSEA at 10 dpi revealed clustering of KEGG terms related to immune signalling (which showed the largest changes in expression levels), among which cytokine–cytokine receptor interactions showed the highest NES (Figure 6A). In contrast to the common early immune responses, terms in the lung-specific group were not enriched for innate immune signalling. Moreover, lung viral loads were not detected after 21 dpi, and the levels of anti-PRRSV antibodies (IgG) started to increase after approximately 10 dpi (Figure 1B). Therefore, based on the viral loads and antibody levels found in this study, we proposed that the immune signalling pathway terms identified in the lung-specific group represent the adaptive immune-response mechanism occurring in the respiratory system.

Many studies regarding the adaptive immune responses that occur during PRRSV were mainly focused on humoral responses associated with various cytokines and the development of cell-mediated immunity (CMI). With regard to humoral responses, it has been reported that non-neutralising antibodies against PRRSV proteins (i.e., the N protein and non-structural proteins) are produced beginning at approximately 5 dpi, whereas neutralising antibodies (NAs) were detected later after approximately 28 dpi [27, 42]. Although PRRSV-specific T cells were previously observed early in lymphoid tissues (beginning at approximately 14 dpi), the T cell responses did not last long and did not correlate with viral loads [43]. In addition, another study showed that the activity of IFN-γ-secreting CD8^+^ T cells against PRRSV was weak and delayed [44, 45]. As previous studies have shown, NAs (humoral responses) and cellular responses (CMI) involved in adaptive immune signalling against PRRSV were detected at abnormally low levels and were delayed, which may reflect the sequential triggering of interference and delayed innate-immune signalling. Lymphoid organs (BLNs and tonsils) were not highlighted in the lung-specific group due to weaker immune responses in these tissues compared to those in the lungs. Significant GO terms related to immune responses and apoptosis were identified in each tissue at 10–35 dpi in the BLN in this study (Additional file 2), but the tonsils tissues were difficult to study in terms of associated mechanisms because of the small number of DEGs. In agreement with the results of this study, a previous study demonstrated that changes in the expression of proinflammatory cytokines (IL-1α, TNF-α, and IL-6) at 3 to 24 dpi in PRRSV (European strain 2982)-infected lymphoid organs (retropharyngeal lymph nodes, mediastinal lymph nodes, and tonsils) were found only in the lymph nodes [46]. Consequently, we discovered that adaptive host immune signalling in response to PRRSV infection was relatively active in the lungs, although such signalling was significantly lower in lymphoid organs (BLNs and tonsils).

### AMPK signalling and lipid metabolism in response to PRRSV infection in BLNs

The BLN-specific group contained clusters 4 and 5 (showing down-regulated genes) and was highly enriched for the AMPK signalling pathway, the PPAR signalling pathway, glycerolipid metabolism, and the adipocytokine signalling pathway, which are related to lipid metabolism (Figure 4D). During viral infection, lipid metabolism can be regulated by viruses; it promotes membrane fusions during viral entry and efficient replication [47, 48]. Among these pathways, several viruses have been reported to inhibit AMPK signalling, which plays a major role in cellular-energy homeostasis including lipid production. Human immunodeficiency virus (HIV) is known to encode a trans-activating regulatory protein (Tat), which has been reported to inhibit phosphorylation of the AMPK α-subunit at Thr172 and to concomitantly reduce the phosphorylation of the AMPK substrate, acetyl-CoA carboxylase [49]. It was also reported that the HCV proteins NS4B and NS5A can activate protein kinase B (PKB/Akt), which inhibits AMPK by phosphorylating Ser485 and Thr172 [50–53]. Moreover, previous reports showed that HIV, HCV, and influenza A virus replication could be inhibited by 5-aminoimidazole-4-carboxamide ribonucleotide (AICAR), which is an AMPK activator [49, 53, 54]. MARC-145 and porcine monocyte-derived dendritic cells (mDCs) infected with PRRSV (North American strain P129-GFP) showed significant suppression by AMPK activators (sodium salicylate and U18666A) [55]. Recently, it was confirmed that AMPK activity increased at up to 1.5 dpi in PK-15^CD163^ cells, that PAMs were infected with PRRSV (North American strain WUH3), that replication was inhibited through acetyl-CoA carboxylase 1 (ACC1), and that fatty acid biosynthesis was reduced by the AMPK activator A769662 [56]. In addition, proteins nsp2, nsp3, and nsp5 of PRRSV are also well known to induce cell membrane rearrangement for efficient replication [12], and it may interact with lipid metabolism such as AMPK signalling. In this study, we performed enrichment analyses for the BLN-specific group in the GCN, which confirmed that genes related to AMPK signalling were only down-regulated in BLN tissues (Figure 3). These results suggest that PRRSV regulates AMPK signalling to create a suitable environment for viral replication in lymphoid tissues including BLNs, which function as a reservoir, thereby establishing persistent infection.

### Host-gene modulations during PRRSV infection

We examined highly significant pathways including influenza A and cytokine–cytokine receptor interactions through enrichment analyses for each group in the GCN construction and GSEA, based on all genes. We also intensively investigated the expression levels of significant genes identified through both analyses.

Firstly, *RSAD2* (Viperin), *DDX58* (RIG-I), *CXCL10* (IP10), *MX1* (MxA), *RNASEL* (RNaseL), *IFNB1* (IFNβ), *IFIH1* (MDA5), *OAS2* (2′-5′OAS and OAS), *IFN-ALPHAOMEGA* (IFNα), *IL6* (IL6), *PML* (PML), and *CCL2* (MCP1) were up-regulated in association with influenza A (Figure 5A). Interestingly, *IFN-ALPHAOMEGA* (IFNα), *IFNB1* (IFNβ), *IL6* (IL6), and *CCL2* (MCP1) were modulated only in lymphoid organs (BLNs and tonsils) (Figure 5B). All selected genes were associated with antiviral and immune signalling. In particular, *RSAD2* (Viperin), *DDX58* (RIG-I), *MX1* (MxA), and *OAS2* (2′-5′OAS and OAS) were highly expressed in all tissues at 3 dpi (log_2_ FC ≥ 2.5), and *IFNB1* (IFNβ) and *IFN-ALPHAOMEGA* (IFNα) were extremely highly expressed at 3 dpi in the lymphoid organs (BLNs and tonsils) (log_2_ FC ≥ 6.0). *RSAD2* (Viperin) encodes a cellular protein that was found to inhibit the replication of various DNA and RNA viruses, including influenza A [57]. During influenza A infection, Viperin decreased lipid raft formation by reducing the activity of farnesyl diphosphate synthase, which inhibited viral budding and release [58]. In addition, Viperin up-regulation in PRRSV (North American strain BB0907)-infected MARC-145 cells was reported to inhibit viral replication [59]. RIG-I, a PRR responsible for type-I interferon responses, is an essential molecule for innate immune signalling that recognises virus-infected cells in mammals, including pigs [60]. The human MxA protein has been shown to exert antiviral activity against a wide range of RNA viruses (including influenza A) and some DNA viruses [61]. Moreover, expression of the porcine Mx1 protein was reported to increase at up to 7 dpi in PRRSV (American strain SNUVR970501)-infected MARC-145 cells [62]. *OAS2* is known to encode a member of the 2-5A synthetase family, which comprises essential proteins involved in the innate immune responses to viral infection; it promotes viral RNA degradation and inhibition of viral replication [63]. Furthermore, it was reported that OAS2 overexpression in PAMs inhibited PRRSV (North American strain BJ-4) replication [64].

*IFN-ALPHAOMEGA* encodes a unique isoform found only in a few species such as pigs and cows [65], and its antiviral activity against PRRSV (North American strain SDSU-23983-P140) infection was found to differ depending on the target cell [66]. In addition, *IFNB1*, one of the representative signalling genes involved in antiviral innate immunity in mammals including pigs, showed high antiviral activity in PAMs, but not in MARC-145 cells, indicating an opposite effect compared to that of *IFN-ALPHAOMEGA* [66]. In this study, we also focused on the top 25 proteins showing high interactions with *IFNB1* through the PPI network (Figure 7), some of which (IFNAR1, IFNAR2, IRF3, IRF7, IRF9, STAT1, and STAT2) were associated with IFN-mediated immune responses (autocrine and paracrine signalling) and promoted IFN secretion through intracellular pathogen recognition [67]. Generally, pathogens are recognised by PRRs, and IFN expression can be induced in the nucleus through the activation and phosphorylation of IRF3 and IRF7. IFNs secreted from cells can bind to the receptors IFNAR1 and IFNAR2, which initiates signal transduction pathways; subsequently, IFN-stimulated genes are expressed after the formation of the ISGF3 complex (IRF9, p-STAT1, and p-STAT2). Collectively, these findings suggest that the host responses at 3 dpi in all tissues were associated with up-regulated genes related to antiviral signalling that were common to the case of influenza A infection, and immune-related genes specifically expressed in lymphoid organs (BLNs and tonsils) were identified. In particular, the *IFN-ALPHAOMEGA* and *IFNB1* genes, which encode type-I interferons (which were expressed at extremely high levels in lymphoid organs), need to be further studied, so as to clarify their molecular functions in PRRSV infection.

Secondly, cytokine–cytokine receptor interactions (confirmed by GCN-enrichment analysis and GSEA) included up-regulated expression of *TNFRSF4* (Ox40), *TNFRSF9* (4-1BB), *TNFRSF6B* (DCR3), *CCL20* (CCL20), *CD70* (CD70), *CXCR3* (CXCR3), *IL20RA* (IL20RA), *TNFSF11* (RANKL), *CCR3* (CCR3), *CCL5* (CCL5), *IL26* (IL26), and *IL2RB* (IL2RB), which include adaptive immune-related genes (Figure 6A). TNFSF and TNFRSF are known to control innate and adaptive immune cells by regulating various mechanisms, inducing the co-stimulation or co-inhibition of immune responses [68]. Ox40 is a co-stimulatory receptor expressed in activated T cells after antigen recognition, and interaction with its ligand can promote T cell proliferation and survival, as well as cytokine production. 4-1BB is an inducible co-stimulatory receptor that is mainly expressed in T cells, and interaction with its ligand is essential for T cell development, survival, proliferation, effector function, and memory T cell formation [68]. RANKL is a strongly up-regulated ligand in T cells after antigen receptor stimulation, and it affects bone homeostasis, lymphoid organ development, and T cell–dendritic cell interactions [68]. In summary, we identified genes affecting T cell maturation, proliferation, and survival with respect to adaptive immune signalling at 10 dpi; however, further studies should be performed to clarify the roles of these genes during PRRSV infection.

### Study limitations, conclusions, and future prospects

A possible concern regarding the generation of serial data for these respiratory and immune tissues is the use of tissue samples obtained from different animals at each time point. However, the trends observed for serum viral loads and antibody titres (Figure 1B) corresponded with those of previous studies [15, 69, 70] that used blood collected from genetically identical pigs from multiple time points, indicating that the pigs used in this study could have had similar responses to PRRSV at any given time point.

We performed DEG profiling at six serial time points with one respiratory and two immunity-responsible tissues during PRRSV infection, based on RNA-Seq data. Additionally, three groups with specific expression patterns (i.e., the 3-dpi, lung, and BLN groups) were discovered by integrating the data via GCN construction. Our findings suggested the involvement of key signalling pathways through functional-enrichment analyses. At 3 dpi, all three tissues showed antiviral and innate-immune signalling similar to the case for influenza A infection, with the lymphoid organs (BLNs and tonsils) showing relatively stronger expression levels in response to infection than the lungs. Moreover, we observed the adaptive immune responses that were most active in the lung tissues, based on high expression levels of various cytokines, whereas the responses were relatively weak in the lymphoid organs. Independently, AMPK signalling appeared to be down-regulated specifically in BLN tissues, resulting in chronic infection through a direct relationship with viral replication. These results provide an understanding of the host’s regulatory mechanisms and should be useful for vaccine development and studying PRRSV resistance.

Furthermore, gene expression can be regulated by epigenetic mechanisms including DNA methylations, histone modifications, and non-coding RNAs. Therefore, we suggest the need for future studies that identify an association between gene expression and epigenetic changes through the integration of multi-omics layers.

## Supplementary information

**Additional file 1. Table S1. RNA quality scores. Table S2. Overview of data processing. Table S3. Interactions of the top 25 proteins in the PPI, based on IFNB1.**

**Additional file 2. GO treemaps were created based on P values for biological process terms specific to the BLN at each time point: (A) 10 dpi, (B) 21 dpi, (C) 28 dpi, and (D) 35 dpi.**

## Data Availability

All data generated or analysed during this study are included in this published article.

## Abbreviations

BLN: bronchial lymph node
BP: biological process
cDNA: complementary DNA
CMI: cell-mediated immunity
DAVID: database for Annotation, Visualization and Integrated Discovery
dpi: days post infection
DEG: differentially expressed gene
FC: fold-change
FDR: false discovery rate
GCN: gene co-expression network
GO: gene ontology
GSEA: gene set-enrichment analysis
HCV: hepatitis C virus
HIV: human immunodeficiency virus
KEGG: Kyoto Encyclopaedia of Genes and Genomes
MDS: multidimensional scaling
NES: normalised enrichment score
PAM: porcine alveolar macrophage
PCIT: partial correlation coefficient with information theory
PPI: protein–protein interaction
PRR: pattern-recognition receptor
PRRS: porcine reproductive and respiratory syndrome
PRRSV: PRRS virus
RLR: RIG-I-like receptor
RNA-seq: RNA sequencing
TCID: tissue culture infectious dose
