# Supplementary figures and images for: Integrated time-serial transcriptome networks reveal common innate and tissue-specific adaptive immune responses to PRRSV infection
Source: Vet Res. 2020 Oct 13;51:128. doi: 10.1186/s13567-020-00850-5 (PMC7552595; doi:10.1186/s13567-020-00850-5)

### Supplementary Figure 1

**(A)**

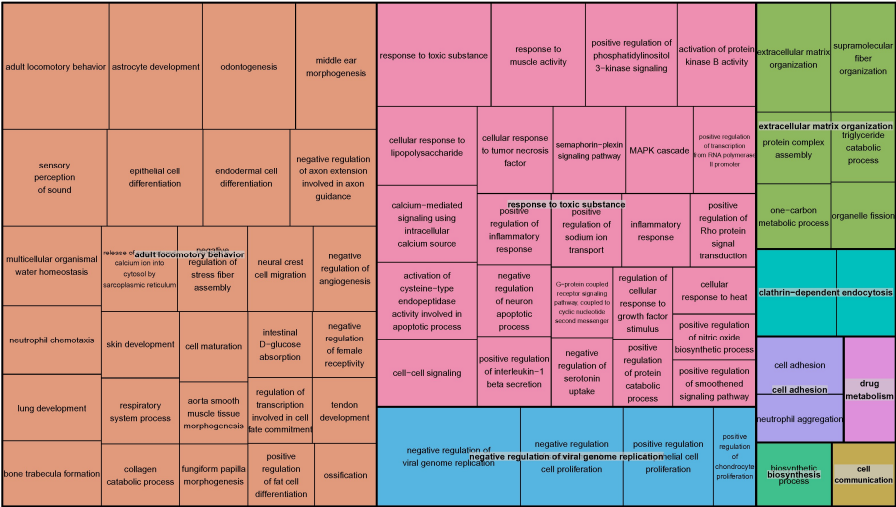

**(B)**

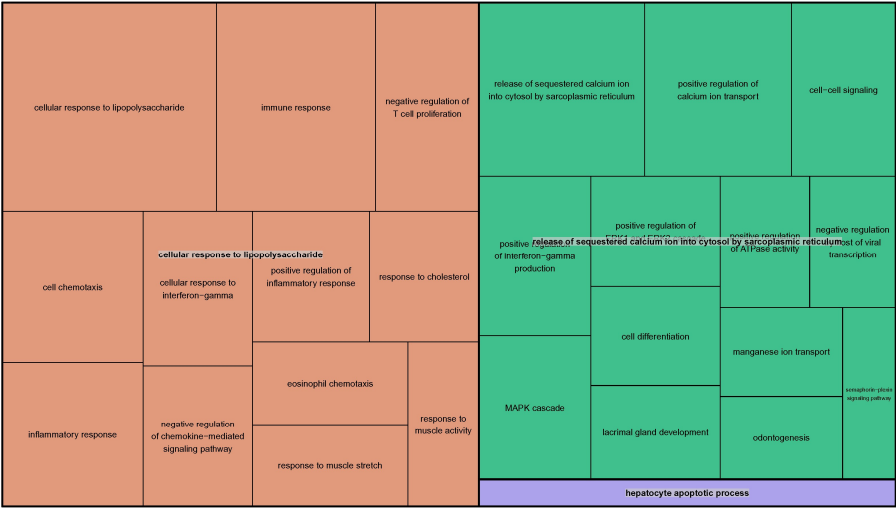

(C)

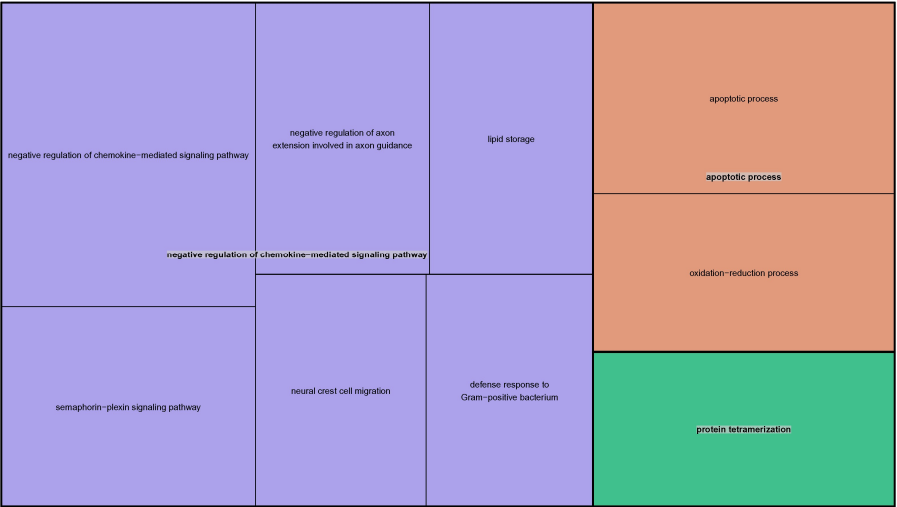

(D)

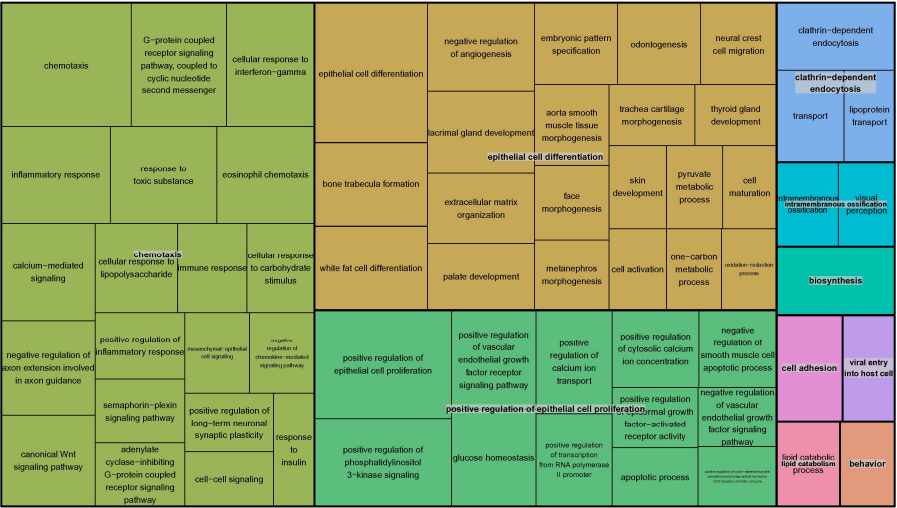

Supplement: Supplementary file 2 — Additional file 2. GO treemaps were created based on P values for biological process terms specific to the BLN at each time point: (A) 10 dpi, (B) 21 dpi, (C) 28 dpi, and (D) 35 dpi. [file 13567_2020_850_MOESM2_ESM.pdf]
